# Ideal and reality: do countries adopt and follow recommended procedures in comprehensive multiyear planning guidelines for national immunization programmes?

**DOI:** 10.1186/s13012-015-0239-8

**Published:** 2015-04-12

**Authors:** Peter Mala, Patrick Zuber, Claudio Politi, Fred Paccaud

**Affiliations:** Faculty of Medicine and Biology, Institute of Preventive and Social Medicine, University of Lausanne, 10 Route de la Corniche, 1010 Lausanne, Switzerland; Department of Essential Medicines and Health Products, World Health Organization, Avenue Appia 20, 1211 Geneva 27, Switzerland; Department of Immunization, Vaccines and Biological, World Health Organization, Avenue Appia 20, 1211 Geneva 27, Switzerland

**Keywords:** Public health guidelines, Adoption and usage, Donor funding incentives, Follow recommendations, Immunization programme planning

## Abstract

**Background:**

Meticulous steps and procedures are proposed in planning guidelines for the development of comprehensive multiyear plans for national immunization programmes. However, we know very little about whether the real-life experience of those who adopt these guidelines involves following these procedures as expected. Are these steps and procedures followed in practice? We examined the adoption and usage of the guidelines in planning national immunization programmes and assessed whether the recommendations in these guidelines are applied as consistently as intended.

**Methods:**

We gathered information from the national comprehensive multiyear plans developed by 77 low-income countries. For each of the 11 components, we examined how each country applied the four recommended steps of situation analysis, problem prioritization, selection of interventions, and selection of indicators. We then conducted an analysis to determine the patterns of alignment of the comprehensive multiyear plans with those four recommended planning steps.

**Results:**

Within the first 3 years following publication of the guidelines, 66 (86%) countries used the tool to develop their comprehensive multiyear plans. The funding conditions attached to the use of these guidelines appeared to influence their rapid adoption and usage. Overall, only 33 (43%) countries fully applied all four recommended planning steps of the guidelines.

**Conclusions:**

Adoption and usage of the guidelines for the development of comprehensive multiyear plans for national immunization programmes were rapid. However, our findings show substantial variation between the proposed planning ideals set out in the guidelines and actual use in practice. A better understanding of factors that influence how recommendations in public health guidelines are applied in practice could contribute to improvements in guidelines design. It could also help adjust strategies used to introduce them into public health programmes, with the ultimate goal of a greater health impact.

## Background

Whether considered as a staged or an iterative process, public health policy and practice involve the use of guidelines in planning and implementing interventions [1,2]. These instruments provide the principles, procedures, and techniques used in virtually every phase of the implementation circle, from initiation to formulation, implementation, and finally, evaluation of the outcomes and impacts [3-7]. Even though systematic effort to convert basic research knowledge to evidence-based guidelines and practice is relatively new in public health compared to clinical medicine [8,9], there is increasing body of scientific literature and research on the use of basic public health science and new discoveries in public health policies and practice guidelines [9-15]. Additionally, instruments such as the Grading of Recommendations Assessment, Development and Evaluation (GRADE) framework are increasingly being used by the public health community to appraise the quality of evidence for public health recommendations and production of public health guidelines [16,17].

The production of public health guidelines and their effective use are two complementary aspects. We know much about the types and number of public health guidelines being produced. For example, the World Health Organization (WHO) alone produces at least 20 public health guidelines annually. These guidelines include the global framework for immunization monitoring and surveillance [18], the updated guidelines for evaluating public health surveillance systems [19], the guidelines for intensified tuberculosis case-finding and isoniazid preventive therapy for people living with HIV in resource-constrained settings [20], a comprehensive multiyear planning tool for immunization programmes [21], and the United Nations Children’s Fund (UNICEF) cholera toolkit [22], among others. However, there is very little information on whether or how these tools are actually used in public health practice in countries with varying political, economic, and social systems [23].

The question of whether these tools are applied as consistently as intended is not often addressed in public health research. Despite this gap in knowledge, the public health community generates and disseminates public health guidelines without, in most instances, critically assessing them and building on the experience gained. Sometimes, the guidelines being released or promoted by different public health agencies are more or less the same, except for minor differences in terminology and certain details. An assessment of how recommendations and procedures of public guidelines are followed in public health programmes is necessary if the instruments are to add value to the health of populations [24,25].

### Policy context for the study

Guidelines for the development of comprehensive multiyear plans (cMYPs) for national immunization programmes were developed by the WHO, UNICEF, and partners to support countries in implementing the Global Immunization Vision and Strategy (GIVS). The GIVS is a global immunizational policy framework that was introduced in 2005 in order to enhance the impact of immunization programmes and further reduce infant mortality due to vaccine-preventable disease, particularly in low-income countries [26,27]. These guidelines represent a new approach to immunization planning, guided by the need to simplify and harmonize a large number of immunization-planning activities at the national level. They aim to assist countries in streamlining the immunization planning process at the national level into a single comprehensive and budgeted plan and to minimize the duplication of efforts and transaction costs for countries and partners [26].

The guidelines are based on the rational model of planning [28,29], which follows a logical process beginning with situation analysis, followed by ranking and prioritization of the problems identified, evaluation and selection of possible interventions, and assessment of performance-monitoring indicators and milestones [21,30,31]. The guidelines have been in use since 2005, and the WHO and other partners have played an active role in facilitating their adoption and use through training workshops [32-34] and technical assistance. Since the instrument was introduced, the GAVI Alliance (Gavi) has required countries that applied for specific windows of funding to support their application with a cMYP developed based on the guidelines [33]. The gross national income (GNI) per capita threshold that was set to establish eligibility for Gavi financial support was US$1,000 between 2000 and 2010; thereafter, it was adjusted to US$1,500 [34].

In this study, we examined the patterns of adoption and alignment of the cMYPs with the recommendations and procedures of the guidelines in national immunization programmes. The purpose of our study is to partially fill the gap in knowledge between recommendations and procedures of public health guidelines and whether they are followed among the target audience in public health practice.

## Methods

A document review was conducted on 77 countries that had adopted the guidelines for the development of cMYPs for national immunization programmes. The WHO financing and planning database [35] was used to identify countries that have developed at least one cMYP based on the guidelines. This database is a repository of copies of cMYPs developed by countries and shared with the WHO. As of December 2012, 77 countries had developed and submitted a copy of at least one cMYP, including 54 that had developed a second cMYP. In total, 77 copies of the first cMYPs were retrieved from the database for review and data extraction.

The detailed data that were extracted from the cMYPs included information on how the planning steps proposed by the guidelines were applied by the countries in each of the 11 national immunization planning components identified. Those planning components include the following 1) vaccine supply and stock management, 2) cold chain, 3) human resources, 4) vaccination implementation, 5) immunization safety, 6) adverse events following immunization (AEFI), 7) waste management, 8) surveillance and laboratory analysis, 9) coordination and supervision, 10) immunization promotion, and 11) government funding.

A descriptive analysis was performed using the extracted data to determine adoption patterns and the extent to which countries followed the recommended planning steps in the guidelines, as the tool was used by different countries in the development of the cMYPs. Alignment of the cMYPs with the recommended planning steps was assessed based on inclusion or omission of the steps, as well as inclusion of a minimum set of information for the included steps. A step was considered followed if a cMYP included the step and also, for the first (situation analysis) step, at least one performance gap was included; for the second (problem prioritization) step, at least one national immunization programme priority was specified; for the third (intervention selection) step, at least one national immunization programme intervention was specified; and for a chosen intervention in the fourth (indicator selection) step, at least one performance indicator was specified.

## Results

**Figure 1:**
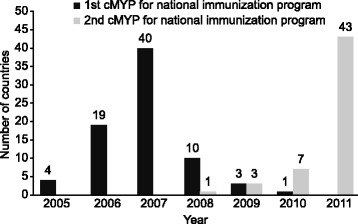
Trend of cMYP development using the guidelines by countries from 2005 to 2011.

Among the 77 countries studied here, there was a rapid increase in the number of countries that adopted the guidelines and developed their first cMYPs based on those guidelines, from 4 (5%) in 2005 to 66 (86%) by the end of 2007. From 2008, 54 (70%) countries that reached the end of the time period covered by their first cMYP developed a second one; only one country developed a second cMYP in 2008 and by 2011, a total of 54 countries had developed a second cMYP using the guidelines (Figure 1).

Since 2005, Gavi-eligible countries have been required to support their applications for specific windows of funding with a cMYP developed using the guidelines. As a result, all 72 (94%) countries that received Gavi support during the period studied developed a cMYP based on the guidelines. As Gavi eligibility is based on GNI per capita, the majority of countries that used the guidelines were low-income countries. Another three countries (4% of those studied) used the guidelines to develop a cMYP, although they were not eligible for Gavi support; those countries include Namibia, Botswana, and Swaziland. The WHO’s African region, with a high proportion of Gavi-eligible, low-income countries, accounted for the largest numbers of countries studied (*N* = 40, 52%) (Table 1), followed by Southeast Asia (*N* = 9, 12%), the Eastern Mediterranean (*N* = 8, 10%), Europe (*N* = 7, 10%), the Western Pacific (*N* = 7, 10%), and the Americas (*N* = 6, 8%). The European, Western Pacific, and American regions, including predominantly high-income countries, had the lowest number of countries that used the guidelines to develop a cMYP.

**Table 1:** **Countries by WHO region developing at least one cMYP using the guidelines**

**Year**	**AFR [** ***n*** **= 46]**	**AMR [** ***n*** **= 35]**	**EMR [** ***n*** **= 22]**	**EUR [** ***n*** **= 53]**	**SEAR [** ***n*** **= 11]**	**WPR [** ***n*** **= 27]**	**Total [** ***n*** **= 194]**
2005	2 (2)	_	1 (1)	_	1 (1)	_	4 (4)
2006	11 (10)	1 (1)	2 (2)	1 (1)	_	4 (4)	19 (18)
2007	20 (19)	4 (3)	4 (4)	4 (4)	5 (5)	2 (2)	39 (37)
2008	5 (4)	_	1	2 (2)	1 (1)	1 (1)	10 (8)
2009	1 (1)	1 (1)	_	_	1 (1)	_	3 (3)
2010	1 (1)	_	_	_	_	_	1 (1)
2011	_	_	_	_	1 (1)	_	1 (1)
Total	40 (37)	6 (5)	8 (7)	7 (7)	9 (9)	7 (7)	77 (72)

Numbers in parentheses indicate the number of countries with a GNI per capita income ≤ US$1,000 that were eligible for Gavi funding.

*Abbreviations*: *AFR* WHO African region, *AMR* WHO region of the Americas, *EMR* WHO Eastern Mediterranean region, *EUR* WHO European region, *SEAR* WHO South East Asia region, *WPR* WHO Western Pacific region.

We examined how rigorously the four planning steps in the guidelines have been applied by the countries that developed a cMYP (Table 2). The first step, situation analysis, was the most followed among the 77 countries that used the guidelines; 70% to 94% of the countries applied the situation analysis step in all 11 planning components of the national immunization programme. The second planning step, problem prioritization, was the second most applied with 65% to 90% of the 77 countries applying the step in 10 of the 11 planning components. In third position was the third planning step, intervention selection, which showed 48% to 88% of the 77 countries applying the step in 10 of the 11 planning components. The fourth planning step, indicator selection, had the lowest percentage of countries that aligned their plans with the step, with 0% to 7% of the countries applying the step in 10 of the 11 planning components.

**Table 2:** **Number of countries that followed the planning steps in the cMYP guidelines**

**Immunization planning component**	**Situation analysis step**	**Problem prioritization step**	**Intervention selection step**	**Indicator selection step**	**Compliance with all four steps**
>60% followed all four planning steps					
1. Human resources	72 (94)	69 (90)	68 (88)	58 (75)	58 (75)
2. Cold chain	69 (90)	69 (90)	61 (79)	55 (71)	55 (71)
3. Vaccination implementation	63 (82)	60 (78)	60 (78)	60 (78)	60 (78)
4. Vaccine supply and stock management	62 (81)	61 (79)	60 (78)	54 (70)	54 (70)
30% to 60% followed all four planning steps					
5. Immunization safety	60 (78)	56 (73)	49 (64)	44 (57)	44 (57)
6. Waste management	59 (77)	57 (74)	40 (52)	31 (40)	31 (40)
7. Government funding	57 (74)	54 (70)	37 (48)	33 (43)	33 (43)
8. AEFI monitoring	56 (73)	50 (65)	44 (57)	25 (32)	25 (32)
<30% followed all four planning steps					
9. Surveillance and laboratory	67 (87)	64 (83)	63 (82)	8 (10)	8 (10)
10. Immunization promotion	58 (75)	54 (70)	50 (65)	0 (0)	0 (0)
11. Coordination and supervision	54 (70)	53 (69)	44 (57)	0 (0)	0 (0)

Numbers in parentheses indicate the percentage of countries implementing the planning step(s).

The extent to which the four planning steps in the guidelines were followed in the cMYPs developed by the 77 countries was further examined based on individual planning components; 3 categories were identified among the 11 planning components (Table 2). The first category consisted of four components with the highest proportion of countries (>60%) that followed all four planning steps, which included the cold chain, human resources, vaccination implementation, and vaccine supply and stock management planning. Vaccination implementation had the highest number of countries that followed all four planning steps in the development of their cMYPs in this category, with 60 (78%) countries applying all four planning steps. The human resources planning component had the second highest number of countries that followed all four planning steps, with 58 (75%) countries applying all four steps, followed by the cold chain planning component, with 55 (71%) countries applying all four planning steps. Finally, 54 (70%) countries applied all four steps in the vaccine supply and stock management planning component in this category.

The second category consisted of four planning components with 30% to 60% of the countries applying all four planning steps of the guidelines. These planning components included government funding, immunization safety, waste management, and AEFI monitoring planning. Immunization safety had the highest number of countries that followed all four planning steps in this category, with 44 (57%) countries applying all four planning steps, followed by government funding, with 33 (43%) countries applying all four planning steps, and waste management, with 31(40%) countries applying all four planning steps. AEFI monitoring was in last position in this group, with 25 (32%) countries applying all four planning steps.

Finally, the third category contained three planning components with <30% of the countries following all four planning steps. These components included surveillance and laboratory analysis, immunization promotion, and coordination and supervision planning. Eight (10%) countries applied all four planning steps of surveillance and laboratory analysis. None of the countries proposed intervention indicators, the fourth planning step, for immunization promotion, or addressed the coordination and supervision planning components in this category.

## Discussion

The main findings show that a total of 77 countries have adopted and used the guidelines for the development of cMYPs for their national immunization programmes. There was rapid adoption of the instrument, with 66 (86%) countries doing so within the first 3 years of its introduction. Use of the guidelines appeared to be sustained, as 54 (70%) countries had already used the instrument to develop a second cMYP to cover a subsequent time period.

Among the countries that adopted the guidelines, 72 (94%) were low-income countries, with a per capita income not exceeding US$1,000. The African region accounted for the highest percentage of countries that used the guidelines (52%), followed by Southeast Asia (12%). Adoption of the guidelines was minimal among countries with higher incomes, given that only five countries with a per capita income > US$1,000 adopted the guidelines and that none of the high-income countries adopted the guidelines.

The findings that we have presented regarding the patterns of adoption and use of the guidelines suggest that the technical merits of the instrument alone were not the only considerations in a country’s decision to adopt and use the guidelines; other factors, such as economic status and donor funding, and particularly the linkage of an infusion of Gavi donor funds with use of the guidelines in the preparation of national immunization plans, appeared to be influential. Between 2005 and 2010, eligible countries with a per capita income not exceeding US$1,000 were required by Gavi to support their funding applications with cMYPs based on the guidelines, which may explain the increase in adoption mainly among countries with the lowest incomes. Furthermore, certain funding windows were only available within a specific period of time, and the rush to secure funds before the windows closed most likely contributed to the observed accelerated use of the guidelines among these countries. These observations are consistent with studies that have demonstrated the influence of fiscal context and financial incentives on the accelerated adoption and use of new health practice guidelines [36-38]. Even though no funding was given as an incentive, the linkage of funding to use of the guidelines in preparation of the national immunization plans may have influenced the decisions of countries to adopt the guidelines among countries that needed to apply for these funds.

As we have indicated, despite the rapid pace of adoption and use of the guidelines for cMYP development by many countries, the recommended planning steps in the guidelines were not systematically applied in the 77 cMYPs analyzed. Situation analysis, the first planning step, was the most applied among the countries. Subsequent planning steps were applied to a lesser extent, with indicator selection step (the fourth planning step) being the least respected.

Even though the alignment of the cMYPs with all four planning steps in the guidelines, from situation analysis to indicator selection, was generally satisfactory, much higher levels of omission of the intervention and indicator selection planning steps were observed in most of the planning components. This result appears to suggest that countries do not regard the choice of immunization programme interventions and performance monitoring very highly. Less emphasis on performance monitoring has been observed in other public health programmes in resource-poor settings [39], which may be explained by the fact that, traditionally, the performance and success of national immunization programmes have primarily been measured in terms of the national vaccination implementation and coverage achieved [40]. This idea is consistent with the findings in this study, which showed that the highest number of countries followed all four planning steps in the vaccination implementation planning component compared with the other ten planning components of national immunization programmes.

An examination of how the planning steps were followed in specific planning components further revealed better alignment with the four planning steps of the planning components that come under direct control of immunization programme managers; over 60% of the countries followed all four steps in these planning component, which included cold chain, human resources, vaccination implementation, and vaccine supply and stock management planning components. Comparatively, alignment of the cMYPs with the planning steps of the guidelines was relatively lower among planning components that are shared with other technical units; these components include surveillance and laboratory analysis, which is a core function of epidemiology and public health laboratories units of the MOH; immunization promotion, which is often shared with health promotion units of the MOH; government funding for immunization, which is a function of the national budgeting office; and waste management, which may depend on the sanitation department. It is not clear whether this result was because immunization programme managers are less committed to, or not as experienced with, planning components that are shared with other technical units. However, planning experience has been shown to influence the quality of planning [41].

## Conclusions

This study reveals some gaps between the recommended ideals of the cMYP guidelines and the application of the recommendations in actual practice. While the main limitation of this analysis was a lack of information on the possible explanatory factors for the observed gaps, ensuring the continued use and consistent application of recommendations and procedures of the guidelines by national immunization programmes calls for further studies to gain a better understanding of the factors contributing to these gaps in the diverse economic, political, and social contexts of the implementing countries. This understanding could contribute to the development of better guidelines, more effective introduction strategies, and ultimately more respect for their recommendations among users for greater public health impact.

## Abbreviations

GRADE: Grading of Recommendations Assessment, Development and Evaluation
WHO: World Health Organization
UNICEF: United Nations Children’s Fund
CMYP: Comprehensive Multiyear Plan
GIVS: Global Immunization Vision and Strategy
Gavi: Gavi alliance
GNI: gross national income
AEFI: adverse events following immunization
AFR: WHO African region
AMR: WHO region of the Americas
EMR: WHO Eastern Mediterranean region
EUR: WHO European region
SEAR: WHO South East Asia region
WPR: WHO Western Pacific region
